# Pulmonary Embolism in an Adolescent With May-Thurner Syndrome: A Case Report

**DOI:** 10.7759/cureus.47025

**Published:** 2023-10-14

**Authors:** Mohammed Alpakra, Sameer M Al-Qahtani, Syed Rayees, Badriah G Alasmari, Mohamed F Bazeed

**Affiliations:** 1 Department of Hematology and Oncology, Armed Forces Hospital Southern Region, Khamis Mushayt, SAU; 2 Department of Paediatrics, Armed Forces Hospital Southern Region, Khamis Mushayt, SAU; 3 Department of Pediatrics, Armed Forces Hospital Southern Region, Khamis Mushayt, SAU; 4 Department of Radiology, Armed Forces Hospital Southern Region, Khamis Mushayt, SAU

**Keywords:** whole exome sequencing, hematology, pediatric, anticoagulants, may thurner syndrome, pulmonary embolism, deep venous thrombosis

## Abstract

May-Thurner syndrome (MTS) is a pathological variant where the left iliac vein is compressed by the right iliac artery. Usually common in females between the third and fourth decades of life, this case report is about MTS in an adolescent girl. The patient was overweight and had a sedentary lifestyle. She developed a sudden onset of unilateral left leg deep venous thrombosis (DVT), leading to low-risk pulmonary embolism (PE) within a week of the symptoms starting. The patient received a heparin infusion for one week, after which she was switched to subcutaneous low-molecular-weight heparin. Apart from the initial high D-dimer, the rest of the thrombophilia workup was unremarkable. The whole exome sequencing (WES) study was negative. An inferior vena cava (IVC) filter was not advised for her due to the small size of the clot and her age. The patient responded well to heparin alone and was discharged home on enoxaparin.

## Introduction

May-Thurner syndrome, also referred to as ‘Cockett syndrome’ and ‘iliac vein compression syndrome,’ is a condition in which there is a compression of the left iliac vein by the right iliac artery onto the lumbar vertebrae, which causes deep vein thrombosis. MTS in children is rarely diagnosed as compared to adults. The incidence of DVT in children is rare, with data ranging from 0.07-0.14 per 10,000 children reported. Often, it is associated with coagulopathy, obesity, long-term immobility, and estrogen-containing medication use such as oral contraceptive pills [1]. The presentation is usually unilateral leg swelling with pain and difficulty walking, suggesting acute DVT [2], which sometimes can acutely progress to pulmonary embolism, as in our case; however, sometimes, patients can remain asymptomatic for a longer time. The diagnosis is usually made by Doppler ultrasound, computed tomography venography (CTV), and magnetic resonance venogram (MRV). The gold standard is venous angiography, but since it is an invasive procedure, it is less preferred [3]. The incidence of pulmonary embolism in the pediatric age group is less than 1:100000. Computed tomographic pulmonary angiography (CTPA) is the standard imaging test for the diagnosis of PE. D-dimer levels and echocardiography should also be performed [4]. A thrombophilic workup should be completed when suspecting DVT, as this will affect the outcome of the patient. MTS is mostly seen in females as compared to males. As seen in systematic reviews and meta-analyses on MTS, the cause in children is mostly multifactorial rather than any one cause being fully responsible [5]. In this report, we present a case of an adolescent girl who presented with DVT, quickly progressing to acute pulmonary embolism, eventually leading to the diagnosis of MTS.

## Case presentation

We present a case of a 13-year-old girl, medical and surgical history-free, who presented to the ER with a complaint of left leg swelling for five days. The pain increased gradually and was not associated with fever or trauma. The patient had no family history of any hematological or chronic disease. There is no recent history of the use of oral contraceptive pills. On examination, she was overweight, with a BMI of 28, conscious, and alert. Systemic examination was unremarkable; the left lower limb was warm, erythematous, tender, and swollen, extending to the left inguinal region; the right lower limb was normal. Both limb pulses were palpable.

Initial investigations showed thrombocytopenia, elevated lactate dehydrogenase (LDH) and D-dimer, anti-nuclear antibodies (ANA) negative, and normal cardiac markers (Table 1).

**Table 1:** Laboratory results of complete blood count, cardiac markers, and D-dimer levels of the patient

Measured entity	Current value	Normal range
Hemoglobin	13.6 g/dl	10.9-15 g/dl
Platelet	65 units 10^9^/L	150-400 units 10^9^/L
Lactate dehydrogenase	354 U/L	122-234 U/L
D-Dimer	19.54 mg/L FEU	<0.35 mg/L FEU
Troponin-I	8.1 ng/ml	8.4-18.3 ng/ml
Creatine kinase	32 U/L	28-170 U/L
Aspartate aminotransferase	17 U/L	14-37U/L

**Figure 1:**
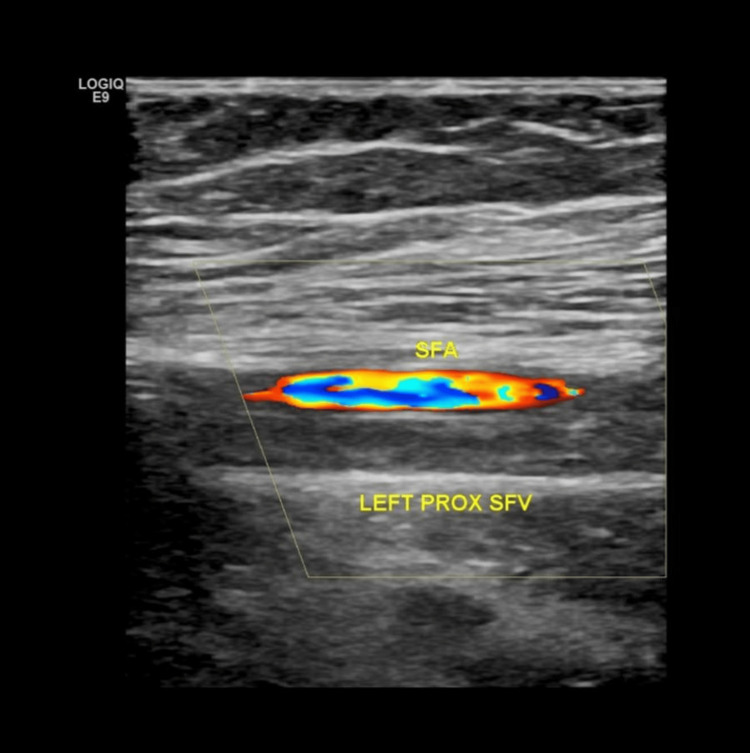
Pre-treatment color Doppler of the left superficial femoral vein

Ultrasound doppler of the left lower limb showed a thrombosed left external Iliac vein, common femoral vein, and superficial femoral vein (proximal and mid), extending through the left saphenofemoral junction to the proximal great saphenous vein (Figure 1). She was admitted to the pediatric ward and was given subcutaneous enoxaparin, and lower limb compression stockings were applied.

**Figure 2:**
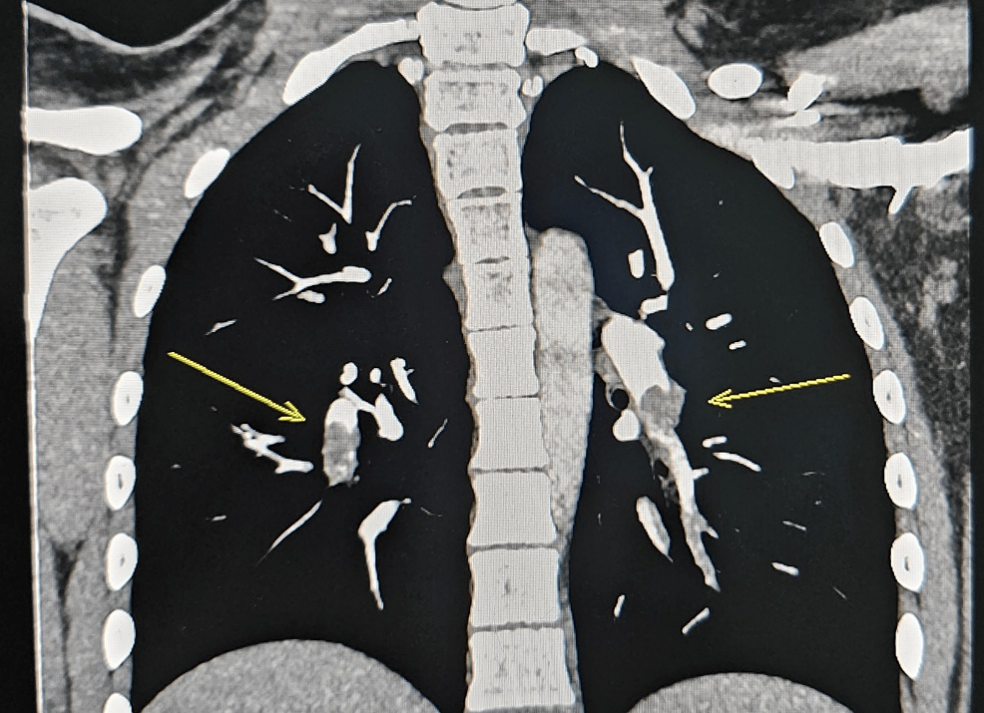
Coronal CT shows filling defects in the pulmonary artery supplying the lower lung lobe bilaterally

On the second day of admission, she became distressed, tachypneic, and had shortness of breath. Urgent CT pulmonary angiography was done, which had the following findings: filling defects are seen within the left main pulmonary artery as well as the basal branches on both sides, representing acute PE (Figure 2). The transthoracic echocardiogram was within normal limits. The ECG showed a normal sinus rhythm. Thereafter, she was shifted to the pediatric intensive care unit and started on heparin infusion with a loading dose of 75 units/kg followed by a maintenance dose of 18 units/kg/hour. The dose was adjusted according to activated partial thromboplastin time (APTT) levels that were monitored every four hours with a target therapeutic range of 50-60 secs.

**Figure 3:**
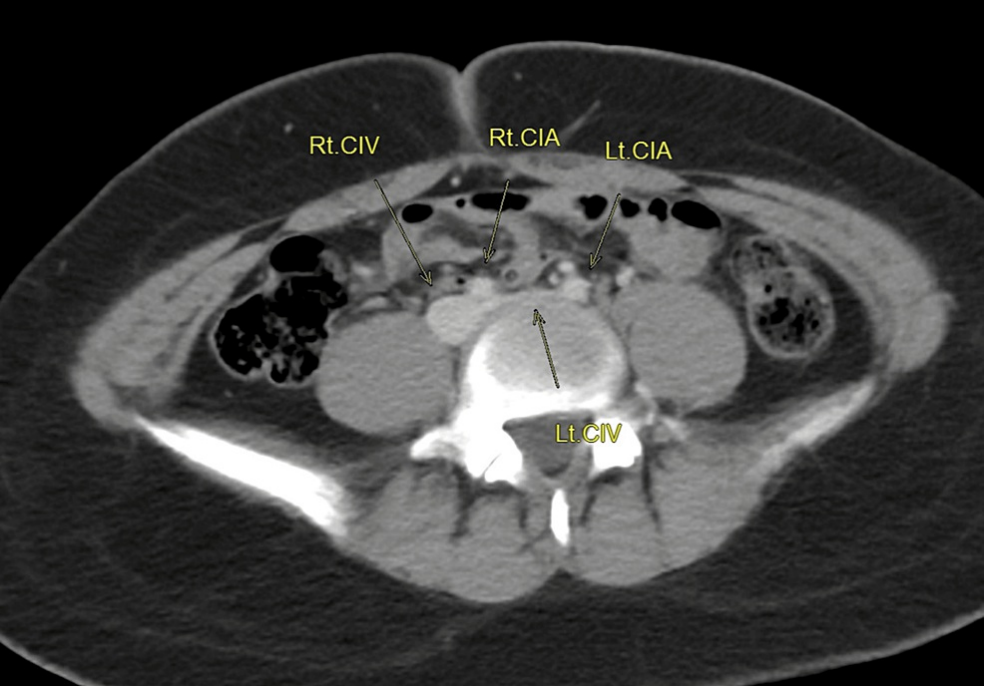
Right and left common iliac arteries markedly compressing the left common iliac vein Rt. CIV: right common iliac vein, Rt. CIA: right common iliac artery, Lt. CIV: left common iliac vein, Lt. CIA: left common iliac artery

CT chest and abdomen with IV contrast was done to localize the extent of thrombosis (Figures 2, 3). It showed that the IVC, right common iliac, external iliac, and internal iliac veins are grossly unremarkable. Dilated, non-enhancing, left common femoral and external iliac veins are thrombosed. The left common iliac vein is seen as non-enhanced and compressed by the right and left common iliac arteries, suggesting a picture of May-Thurner syndrome. Hypercoagulable workup (protein C, protein S, antithrombin III levels, factor V Leiden mutation, homocysteine levels, anticardiolipin antibody, antiphospholipid antibody, and lupus anticoagulant) were within normal limits. The whole exome sequencing (WES) study was negative for congenital thrombophilia. Interventional radiologist opinion was sought for IVC filter, and they suggested that the patient could be a candidate for IVC filter placement at the age of 18 years if clinically indicated at that time.

The patient received a heparin infusion for one week, then switched to subcutaneous low-molecular-weight heparin (LMWH), and was transferred back to the pediatric ward. CBC and coagulation were monitored daily throughout her hospital stay. She was discharged home on the tenth day with good improvement in swelling and pain on subcutaneous enoxaparin.

**Figure 4:**
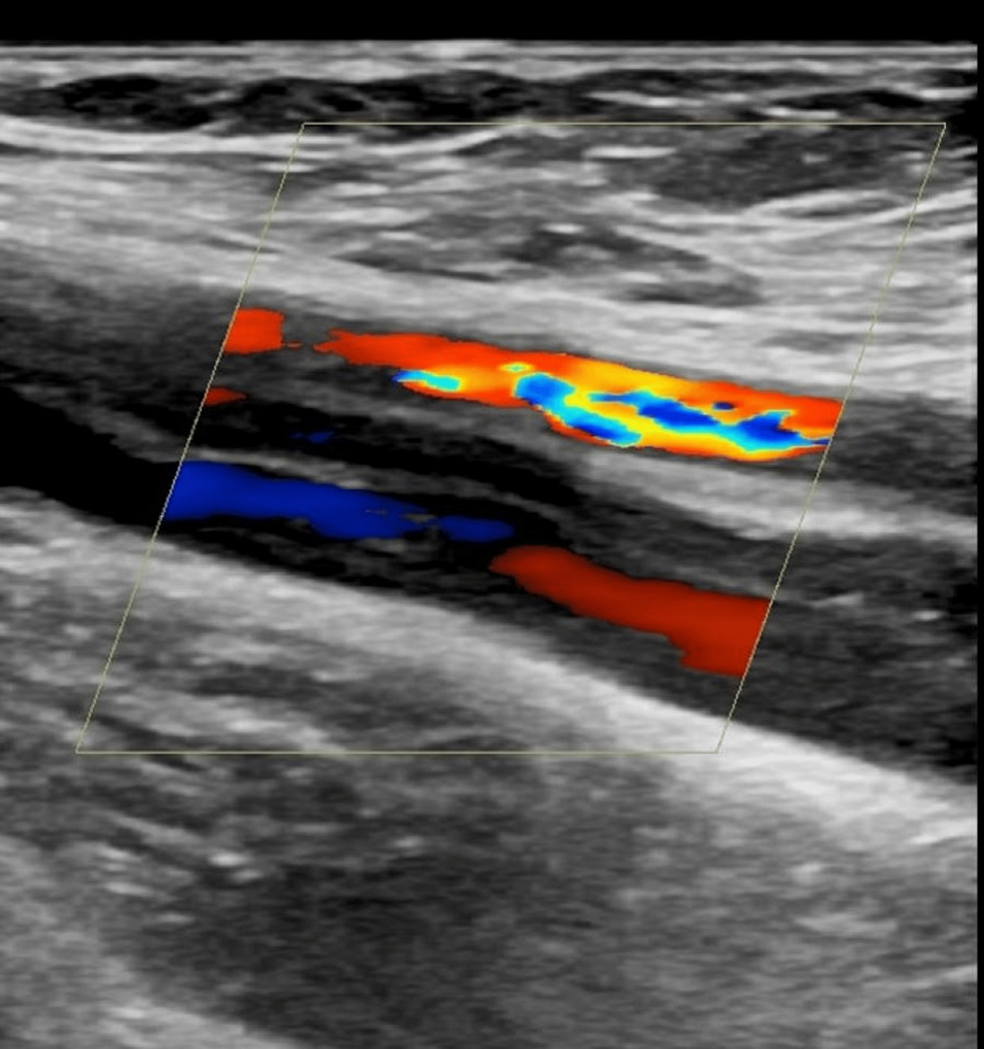
Colour Doppler of the left superficial femoral vein: partially canalized vein after treatment

The patient was admitted electively after one month to a repeat CT of the chest, abdomen, and pelvis, which showed a resolved pulmonary embolism and a tiny filling defect in the IVC at the point of joining the left common iliac vein to the right common iliac vein. A small thrombus or totally collapsed left common iliac vein is seen being compressed by the right and left common iliac arteries. The dilated left external iliac vein and a related part of the common iliac vein with peripheral contrast enhancement and a central filling defect were partially canalized. An ultrasound Doppler repeated at this time showed a partially canalized left superficial femoral vein (Figure 4).

Investigations done at the time of elective admission showed normal platelet count and D-dimer levels. Platelets of 436 units 10^9/L and D-dimer of 0.94 mg/L fibrinogen equivalent units (FEU). She was discharged home with instructions regarding bleeding and advised to continue enoxaparin for six months with regular follow-ups in the hematology and vascular surgery clinic.

## Discussion

MTS is not an inherited or genetic disease. It is an anatomical or mechanical problem that due to hypercoagulable conditions or other risk factors leads to the onset of DVT [2]. In MTS, there is chronic compression of the left iliac vein by the right iliac artery, causing endothelial irritation and collagen deposition, leading to partial or full occlusion of the vein and the formation of deep venous thrombosis [2]. In some patients, this thrombus can migrate to the lungs and cause a pulmonary embolism. Virchow discovered that the left lower extremity was more prone to thrombosis than the right lower extremity in 1851; in 1957, May and Thurner studied 430 cadavers and found a quarter of them had a “spur” formation where the right common iliac artery crossed the left common iliac vein. This spur in the vein eventually leads to wall thickening, injury, and thrombosis [3].

The major risk factors for MTS are hypercoagulable conditions like inherited and acquired thrombophilia, pregnancy and postpartum, surgery, cancer, immobility, obesity, and the use of oral contraceptive pills. As reported in several published studies, MTS in pediatrics is multifactorial. The majority of the patients have more than one cause leading to the development of DVT and, consequently, pulmonary embolism [5]. Most patients are asymptomatic unless triggered. Ninety percent of emboli causing pulmonary embolism are from deep venous thrombosis in the lower limb. Therefore, all risk factors for PE are the same as those for DVT [4].

The diagnosis of MTS is made by imaging. The first line is the ultrasound Doppler, which helps establish the diagnosis in the emergency department. However, iliac vein compression is difficult to assess by ultrasound. Computed tomography (CT) has higher sensitivity. MRV is also helpful but expensive. CTV and MRV identify the thrombus by showing the filling defects caused by contrast [6]. The gold standard test is intravascular ultrasound, but it is less preferred as it is invasive and expensive. Whenever suspecting MTS, all thrombophilic workups should be done to look for any underlying cause. CTPA is the preferred test of choice for diagnosing PE [4].

The main management of MTS is anticoagulants like unfractionated heparin (UH) and low-molecular-weight heparin (LMWH) to prevent further thrombi formation. Pharmaco-mechanical thrombectomy or catheter-directed thrombolysis is done to dissolve the thrombus, after which a stent is placed at the site of compression [2].

MTS usually presents with limb swelling, redness, tenderness, pain, and ulcers, which signify DVT. Shortness of breath, respiratory distress, cough, and hemoptysis indicate the development of PE. The main complication of MTS is post-thrombotic syndrome (PTS), which is chronic leg pain, edema, skin changes, and venous ulcers [7].

In our case, the patient did not have any precipitating factors to trigger MTS. She had a sudden onset of DVT, progressing to PE within a very short period of time. When compared to other published case reports of MTS in pediatrics, our patient had no significant family, medical, or surgical past history. Also, the cause of MTS was more than one risk factor in many published case reports and systematic review and meta-analysis articles [5]. However, there was nothing striking about our patient apart from being overweight and having a sedentary lifestyle. She responded well to conservative management alone, without the need for interventional radiology procedures. Immediate action by other subspecialties leads to a faster and smoother recovery.

## Conclusions

It is essential to consider MTS in the differential diagnosis of DVT and PE in young women. An urgent evaluation of PE and lower extremity DVT for MTS, like in our case, will change the outcome of the patient. The prognosis and response to MTS are very good if treatment is started early with anticoagulants, especially in the pediatric age group. The patient should be in regular follow-up as long as they are on anticoagulant medication. Awareness should be made about the side effects of anticoagulants, with clear instructions for bleeding symptoms and/or breathing difficulty to come any time to the emergency department. The patient and the parents should be educated about the importance of weight management and having an active lifestyle.
